# Study of Knowledge, Attitude, and Practice of Organ Donation Among Medical Students in a Tertiary Care Centre in South India

**DOI:** 10.7759/cureus.4896

**Published:** 2019-06-13

**Authors:** Danny Darlington, Fatima Shirly Anitha, Carbin Joseph

**Affiliations:** 1 Urology, Pondicherry Institute of Medical Sciences, Pondicherry, IND; 2 Pediatrics, Church of South India Kalyani Multispeciality Hospital, Chennai, IND; 3 General Surgery, Government Kanyakumari Medical College and Hospital, Nagercoil, IND

**Keywords:** brain death, deceased donor, organ donation, renal transplantation

## Abstract

Introduction

The deceased donor renal transplantation (DDRT) program in India has seen its ups and downs. The Indian state of Tamilnadu runs a successful DDRT program. Future doctors play an important role in continuing with this success and hence educating them on organ donation is of paramount importance.

Methods

We conducted a cross-sectional questionnaire-based study from June 2015 to December 2017 among 480 medical students to analyse their knowledge, attitude and practice regarding organ donation. The validated questionnaire sheets were distributed during lecture hours and completed sheets were analysed.

Results

Of the 480 participants, 425 completed the questionnaire which is a response rate of 88.5%. Knowledge scores were uniformly low among all four batches of students (p=0.001). The first and third-year students scored better in practice (p=0.001) and attitude (p=0.001) domains. Females outnumbered males by scoring high in all three domains.

Conclusion

The poor knowledge score among all the batches of medical students is alarming. This implies the need for urgent changes in the medical curriculum to better educate future doctors of the country. Durable changes in practice can be brought about by changing the attitude of medical students.

## Introduction

Transplantation is the treatment of choice for end-stage organ disease as it provides a better quality of life and long-term survival to recipients. However, the shortage of organs and donors is the main hurdle in transplantation. India, in particular, has a huge shortage of organs and donors [1]. Deceased donor renal transplantation (DDRT) helps to bridge the gap of organ shortage as several organs including heart, lungs, liver, kidney, cornea and skin can be retrieved from a single deceased donor. Dialysis is an option for renal replacement therapy in chronic kidney disease however similar replacement therapy cannot be provided for liver or cardiac failure patients. Long waiting lists for transplantation mean that most patients with end-stage organ disease die even before organs are available. India has a deceased organ donation the rate of 0.05-0.08/million population compared to more than 20/million in countries like the United States and Spain [2]. Thus, there is an urgent need to promote DDRT in India. Social stigma, ignorance and illiteracy account for the majority of hurdles in organ donation in India. Several studies report poor knowledge of both the common man and the health care professionals (HCPs) on deceased organ donation and hence long-term improvement in organ donation rates can only be achieved by educating and motivating the people and HCPs. Though at present Tamilnadu runs a successful DDRT program among the Indian states, without proper education on organ donation, these results will be short-lived [3-4]. Studies on knowledge, attitude and practice regarding organ donation are less from Tamilnadu population and hence we conducted this study in Kanyakumari one of the most educated districts in the state of Tamilnadu.

## Materials and methods

Our study aimed to analyse the awareness on organ donation among medical students in a tertiary care hospital. It was a cross-sectional questionnaire based study done between June 2015 and December 2017 among 480 undergraduate medical students of Government Kanyakumari Medical College and Hospital, Kanyakumari, Tamilnadu, India. The first, second, third and fourth year medical undergraduate students participated in the study. The participation was voluntary and details of the participants were kept anonymous. The study was approved by the Institutional Ethics Committee.

A validated self-administered questionnaire was provided to the medical students [5-8]. It consisted of three sections apart from questions on demographics of study participants like age, gender, religion, marital status and year of study in medical school. The first (questions 1 to 13), second (questions 14 to 24) and third (questions 25 to 27) sections assessed the levels of knowledge, attitude and practice habits on organ donation, respectively (Table 1). The responses were recorded on a dichotomous scale as yes or no and the total score obtained was calculated. Higher scores indicate a higher level of knowledge, positive attitude and good practice habits towards organ donation. Scores above or equal to 50% of the maximum score were categorised as high scores whereas those which fell below the 50% limit were categorised as low scores.

**Table 1:** The validated self-administered questionnaire used in the study

Question		Score	
		Yes	No
1.	Have you heard of the term organ donation?	1	0
2.	Have you heard of the term organ transplantation?	1	0
3.	Are you aware of the transplantation of human organs act?	1	0
4.	Do you know where to obtain organ donation cards?	1	0
5.	Can a brain dead patient’s organs be donated?	1	0
6.	Will a certified brain dead registered organ donor be immedi­ately disconnected from ventilation support?	0	1
7.	Can parents or guardians make substitute decision making for mentally disabled persons in the regard of organ donation?	1	0
8.	Donor’s and recipient’s blood group must be matched?	1	0
9.	Donor’s human leukocytes antigen must be identical to that of the recipient for any organ transplantation?	0	1
10.	Hepatitis b and c carriers can donate all of their solid organs except the liver organs?	0	1
11.	Malignancy is always a contraindication to cadaveric organ do­nation?	1	0
12.	Increased risk of opportunistic infections is a common compli­cation to all transplantations?	1	0
13.	Organ transplant recipients are more prone to developing of cancer after transplantation?	1	0
14.	Do you support organ donation?	1	0
15.	Do you feel comfortable to think or talk about organ donation?	1	0
16.	Do you agree to donate organs when you die?	1	0
17.	Do you agree to donate your family member’s organs?	1	0
18.	Does your family agree with organ donation?	1	0
19.	Do you think donating one’s organ adds meaning to one’s life?	1	0
20.	Does your religion agree with organ donation or transplanta­tion?	1	0
21.	Do you have belief that your body should be kept intact after death?	0	1
22.	Do you have fear that your body will be disfigured, if you do­nate organs?	0	1
23.	Do you think there will be premature termination of medical treatment for registered organ donors?	0	1
24.	Do you think live organ donation is better than cadaveric organ donation in solving shortage?	1	0
25.	Have you pledged/signed to donate an organ?	1	0
26.	Have you ever donated an organ?	1	0
27.	Did you ever receive an organ for transplantation?	1	0

The questionnaire was distributed to medical students during the lecture hours. They were given instructions to avoid discussing about the questions and to choose their own answers from the given options. The completed questionnaire sheets were collected and analysed. The data were entered into a Microsoft Excel sheet and analyzed using SPSS software (2008, Version 17.0, SPSS Inc., Chicago, USA). The discrete variables were analysed using the Chi-square test and p value less than 0.05 was considered statistically significant.

## Results

**Figure 1:**
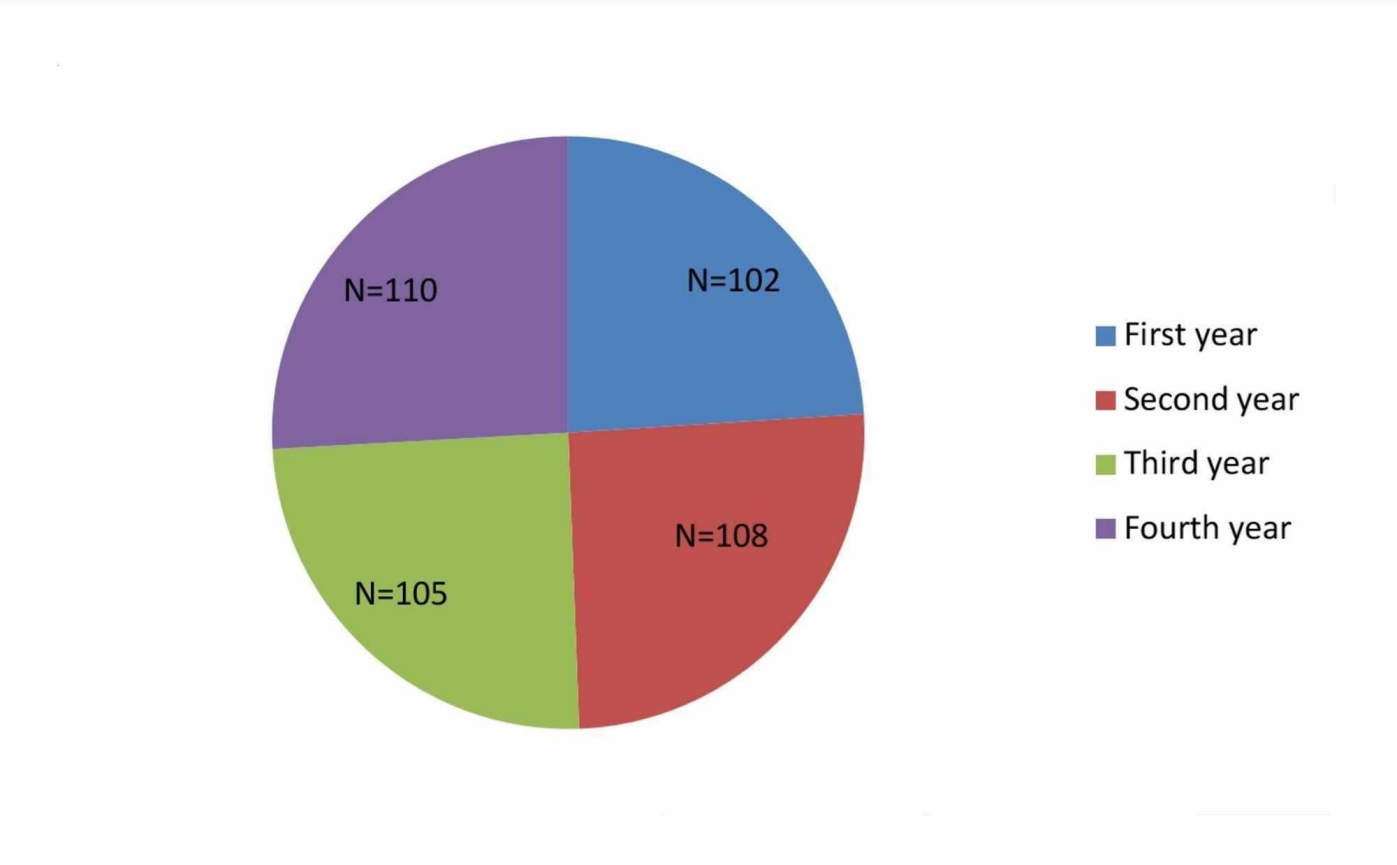
Pie chart showing the year-wise distribution of medical students who completed the study questionnaire N = number of students

Out of the 480 participants, 425 completed the questionnaire which is a response rate of 88.5% (Figure 1). The study population consisted of 260 (61.17%) females and 165 (38.82%) males and all of them were unmarried. Majority of the study population were Hindus comprising 64.17% and the remaining were Christians and Muslims comprising 21.65% and 17.18%, respectively. The response rate was highest among the first year students (96%) and least among the fourth year students (80%).

The knowledge scores were uniformly lower among all the four batches of medical students. Positive attitude towards organ donation was shown by more of the first and third year students than other batches (p=0.001). Similarly more first and third year students scored high in the practice section of the questionnaire than the second and fourth year batches (p=0.001) (Table 2).

**Table 2:** Year-wise comparison of knowledge, attitude, and practice scores of the study participants N = number of students *chi square test

Knowledge	Low Score (˂50% of maximum score) N (%)	High Score (≥50% of maximum score) N (%)	p value
First year	77 (75.49)	25 (24.51)	0.001^*^
Second year	56 (51.80)	52 (49.20)	
Third year	68 (64.76)	37 (35.24)	
Fourth year	80 (72.72)	30 (27.28)	
Attitude			0.001^*^
First year	39 (38.23)	63 (61.77)	
Second year	75 (69.44)	33 (30.56)	
Third year	35 (33.33)	70 (66.66)	
Fourth year	70 (63.63)	40 (36.37)	
Practice			0.001^*^
First year	31 (30.40)	71 (69.60)	
Second year	80 (74.07)	28 (25.93)	
Third year	34 (32.38)	71 (67.62)	
Fourth year	56 (50.90)	54 (49.10)	

More number of Muslims and Christians had high scores than Hindus when attitude was assessed (p=0.001) while the practice scores of Hindus and Muslims were statistically better than Christians (p=0.031) (Table 3). In terms of high scores, female medical students significantly outnumbered males in all three domains as assessed by the study questionnaire (Table 4).

**Table 3:** Religion-wise comparison of knowledge, attitude, and practice scores of study participants N = number of students *Chi-square test

Knowledge	Low Score (˂50% of maximum score) N (%)	High Score (≥50% of maximum score) N (%)	p value
Hinduism	118 (45.38)	142 (54.62)	0.531^*^
Christianity	48 (52.17)	44 (47.83)	
Islam	34 (46.57)	39 (53.43)	
Attitude			0.001^*^
Hinduism	187 (71.92)	73 (28.08)	
Christianity	32 (34.78)	60 (65.22)	
Islam	32 (43.83)	41 (56.17)	
Practice			0.031^*^
Hinduism	114 (43.84)	146 (56.16)	
Christianity	55 (59.78)	37 (40.22)	
Islam	35 (47.94)	38 (52.06)	

**Table 4:** Gender-wise comparison of knowledge, attitude, and practice scores of the study participants N = number of students *Chi-square test

Knowledge	Low Score (˂50% of maximum score) N (%)	High Score (≥50% of maximum score) N (%)	p value
Male	108 (65.45)	57 (34.55)	0.020^*^
Female	196 (75.38)	64 (24.62)	
Attitude			0.004^*^
Male	78 (47.27)	87 (52.73)	
Female	168 (64.62)	92 (35.38)	
Practice			0.001^*^
Male	58 (35.15)	107 (64.85)	
Female	43 (16.53)	217 (83.47)	

## Discussion

Organ donation after brain death has received tremendous promotion in India to sustain the ever-growing demand for organs [4]. The state of Tamilnadu runs a successful transplant program which caters to both national as well as international patients [1]. However, the HCPs play an enormous role in the transplant program. Their role from educating the donors and recipients in providing surgical and medical care to patients is indispensable. The long term success of any transplant program depends on the knowledge and attitude of people it caters to and hence HCPs have an important task to educate and motivate the people. This can be achieved only if the HCPs are well educated about organ transplantation and brain death declaration. Hence, we conducted this study to assess the knowledge attitude and practice habits on organ transplantation among medical students.

The transplantation of human organ (THO) act was passed in the year 1994 in India. It was a major step to improve organ transplantation in India [2]. The state of Tamilnadu runs a successful transplant program which has been refined then and there by several legislative measures. However, lack of proper education negatively influences organ donation. In our study, there is a huge lack of knowledge among medical students on organ donation which needs to be addressed urgently. A study by Patthi et al. pointed out a huge gap in knowledge about organ donation and transplantation among Indian public and medical students [9]. The study by Bharambe et al. also uncovered the same [10]. This gap can be bridged by introducing radical changes in the medical curriculum and the intelligent use of social media [11-12].

Conducting regular camps and seminars for medical students can impart knowledge on organ donation and transplantation. Another approach to promote organ donation is by prompt identification of suitable brain dead donors which requires trained intensivists [13]. Physicians actively involved in treating critically ill patients can be oriented to diagnose brain death through frequent seminars and short courses.

An Indian study by Panwar et al. identified that only 1.4% of subjects willing to donate organs actually registered their names for organ donation [14]. Lack of motivation, lack of faith in the healthcare system, fear of disfigurement, lack of incentives, fear of procedural delays and inappropriate counselling accounted for this attrition. The decision to donate is also influenced by family members and religious leaders. Educational qualification above matriculation was found to positively influence organ donation. In our study, however, knowledge was poor even at the medical school level and this can be rectified by a prompt change in the medical curriculum.

Gender of a person affects his or her decision to donate organs. Men being the main source of income may be reluctant to donate whereas women’s decision to donate is heavily influenced by their parents and spouse. In our study, females fared worse than males in knowledge and attitude but they fared better than males in practice scores. This calls for the education of the female child as she is the backbone of the typical Indian family.

Religious belief has not been found to impact an individual’s decision to donate organs in several studies from India [7-15]. However, in our study more number of Hindus had high practice scores while Christians scored more in a positive attitude towards organ donation. These results are significant considering that the study is done among medical students. In terms of year of study in medical school, first and third year students scored better than other students in practice and attitude scores. This could be partly explained by the eagerness of the first year students to learn new information. Hence, these students can be the prime targets of education on organ donation and transplantation in the future.

The merits of the present study are that it was conducted on a large number of medical school students with high response rates. However, the questionnaire-based nature of the study and its single centre data are the limitations of our study.

## Conclusions

India faces scarcity among plenty as far as organ donation is concerned. The ever-increasing demand for organs can only be met by a multidisciplinary approach to educate the masses. HCPs must be educated about diagnosing brain death and this requires changes in medical curriculum. Unless long-term measures are taken to bring about changes in knowledge, attitude and practice of people and medical professionals the apparent self sufficiency of organs in India will only be short lived.

## References

[1] Abraham G, Vijayan M, Gopalakrishnan N (2016). State of deceased donor transplantation in India: a model for developing countries around the world. World J Transplant.

[2] Shroff S (2009). Legal and ethical aspects of organ donation and transplantation. Indian J Urol.

[3] Shroff S, Rao S, Kurian G, Suresh S (2007). Organ donation and transplantation-the Chennai experience in India. Transplant Proc.

[4] Sachdeva S (2017). Organ donation in India: scarcity in abundance. Indian J Public Health.

[5] Coad L, Carter N, Ling J (2013). Attitudes of young adults from the UK towards organ donation and transplantation. Transplant Res.

[6] Chung CKY, Ng CWK, Li JYC (2008). Attitudes, knowledge, and actions with regard to organ donation among Hong Kong medical students. Hong Kong Med J.

[7] Chakradhar K, Doshi D, Srikanth Reddy B, Kulkarni S, Padma Reddy M, Sruthi Reddy S (2016). Knowledge, attitude and practice regarding organ donation among Indian dental students. Int J Organ Transplant Med.

[8] Sam N, Ganesh R, Indrapriyadarshini V, Jeyamarthan S, Nandhini CK (2018). Awareness, knowledge, and attitude regarding organ donation among final year students of medical, dental, engineering and arts and science colleges in Thiruvallur and Chennai city, India. Indian J Transplant.

[9] Patthi B, Singh S, Singh K, Singla A, Jain S, Kundu H (2015). Beliefs and barriers for organ donation and influence of educational intervention on dental students: a questionnaire study. J Indian Assoc Public Health Dent.

[10] Bharambe VK, Arole VU, Puranam V, Kulkarni PP, Kulkarni PB (2018). Knowledge and attitude toward organ donation among people in Lanja: A rural town in India. Saudi J Kidney Dis Transplant.

[11] Sindhu A, Ramakrishnan TS, Khera A, Singh G (2017). A study to assess the knowledge of medical students regarding organ donation in a selected college of western Maharashtra. Med J DY Patil Univ.

[12] Adithyan GS, Mariappan M, Nayana KB (2017). A study on knowledge and attitude about organ donation among medical students in Kerala. Indian J Transplant.

[13] Palaniswamy V, Sadhasivam S, Selvakumaran C, Jayabal P, Ananth SR (2016). Organ donation after brain death in India: a trained intensivist is the key to success. Indian J Crit Care Med.

[14] Panwar R, Pal S, Dash NR, Sahni P, Vij A, Misra MC (2016). Why are we poor organ donors: a survey focusing on attitudes of the lay public from northern india. J Clin Exp Hepatol.

[15] Bapat U, Kedlaya PG, Gokulnath Gokulnath (2010). Organ donation, awareness, attitudes and beliefs among post graduate medical students. Saudi J Kidney Dis Transplant.

